# Low cost MEMS accelerometer and microphone based condition monitoring sensor, with LoRa and Bluetooth Low Energy radio

**DOI:** 10.1016/j.ohx.2024.e00525

**Published:** 2024-03-26

**Authors:** Morten Opprud Jakobsen

**Affiliations:** Aarhus University, Department of Electrical and Computer Engineering, Finlandsgade 22, Aarhus N, 8200, Denmark

**Keywords:** Mems accelerometer, Mems microphone, LoRa, BLE, Wireless

## Abstract

Vibration-based Condition Monitoring (CM) is an essential tool for identifying potential defects in industrial machinery. However, the implementation of an efficient CM system often necessitates the use of high-cost accelerometers with a large bandwidth. To address this challenge, this study introduces a low-cost CM sensor composed of an ultrasonic MEMS microphone - SPH0641LU and an ADXL1002 MEMS accelerometer. The combination of these two sensor types allows for comparative analysis of the captured data. The SPH641LU microphone is capable of detecting audible vibration signals with a frequency range up to 80 kHz, while the ADXL1002 accelerometer can measure vibrations up to 21 kHz. In addition a three axis ultra low power accelerometer is included, allowing measurement of unbalance or rotating speed, below 200Hz. Moreover, the sensor is designed to operate on battery power and provides the capability for raw data transmission via Bluetooth Low Energy (BLE) or the transmission of pre-processed features from the raw data using LoRaWAN.

## Graphical abstract

**Figure:**
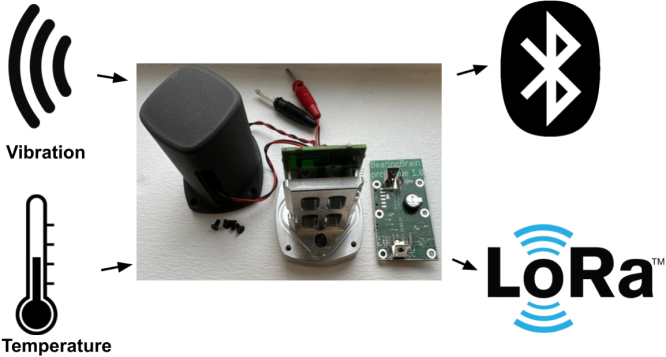

## 1 Specifications table

**Table:** 

**Hardware name**	Low Cost MEMS Accelerometer and Microphone based condition monitoring sensor, with LoRa and Bluetooth Low Energy radio

**Subject area**	•Educational tools and open source alternatives to existing infrastructure • Low cost alternative

**Hardware type**	Vibration monitoring

**Closest commercial analog**	Augury Halo [1]

**Open source license**	Creative Commons Attribution-ShareAlike license

**Cost of hardware**	*180 $ **(Bill of materials)***

**Source file repository**	https://doi.org/10.17605/OSF.IO/KYR59

## 2 Hardware in context

Accelerometer-based condition monitoring systems are widely used for monitoring the health of industrial machinery and detecting faults in real-time. However, in contrast to low cost accelerometers like the ones found in smartphones, fitness trackers and other motion triggered devices, condition monitoring systems require acceleration sensors with larger bandwidths and good signal to noise-ratio to accurately detect and analyze the vibrations emitted by operating machines. The required bandwidth of an accelerometer are typically in the range of tens of kilohertz, as early defects in rotating machines only generate very weak vibration signatures. Evolving defects in most rotating machines only have very limited mechanical impact caused by excitation of eigenfrequencies during infancy [2], [3]. As defects evolve, the severity of impacts increase, and the eigenfrequencies excited during infancy will diminish, and be superseded by the over rolling frequency of the rolling elements, which may often be an order of magnitude lower than the eigenfrequencies excited when a defect is initiated. For a MEMS accelerometer, to obtain the required bandwidth and level of accuracy, often laser trimmed and devices are used. These devices may provide signal to noise ratios and bandwidth comparable to traditional piezo-based sensors [4] but at a higher cost than mass produced non-trimmed MEMS devices.

MEMS microphones are low-cost and up to an order of magnitude cheaper than MEMS accelerometers, when a measurement bandwidth above a few kilohertz is required [5]. This makes them an attractive alternative, or a possible supplement to accelerometers. Acoustic based measurements are subject to interference from background noise, as well as the measurements are sensitive to the distance from source to microphone, but does allow a non-contact install, that may simplify usage [6]. Recently the emerge of acoustic datasets such as MIMII [7] captured from industrial machines and ToyADMOS [8] captured from toys with gears and motors, both running in normal and abnormal conditions has been paving the way for research within acoustic anomaly detection on machines, like the Detection and Classification of Anomalous Sounds and Events forum [9]. The bandwidth given by ultrasonic microphones may also help fill the gap between accelerometers and acoustic emission sensors, that measure beyond 100kHz. Acoustic Emission sensors are capable of measuring stress waves generated by friction and wear [10], [11], but are very sensitive and has severe attenuation across mechanical interfaces [12], and as a result successful installation is difficult and error prone [13].

Several commercial low cost condition monitoring systems exists, such as Amazons Monitron [14], Schaefflers Optime [15], ABBs Ability [16], Augurys Halo [17] who are all offering low cost wireless sensor for condition monitoring. Amazon, ABB and Schaeffler’s devices are performing the bulk of analysis on device, where Augury devices transmit raw data through a gateway for processing. Common for the commercial solutions is that no raw sensor data is exposed or available to the user. A number of open source projects, primarily utilizing accelerometers exists, like Memsio [18], wizfi [19] also exists. Commercial available sensor evaluation platforms, like Infineons Xensiv [20], ST’s Proteus [21], Analog device’s Voyager3 [22] also exists, allowing its users to evaluate individual sensors, and get raw sensor data of the device. The iComox [23] kit combines accelerometers and microphone and a 2.4 GHz mesh radio into a single device for developing condition monitoring systems.

The higher cost and limited bandwidth for accelerometers, presents a potential for using lower cost MEMS microphones for condition monitoring systems, despite their limitations. In this study, we demonstrate the viability of using ultrasound MEMS microphones for condition monitoring as an alternative or a supplement to traditional accelerometer-based systems. A prototype sensor is build, featuring an ultrasound MEMS microphone and a good MEMS accelerometer. This allows the reader to build, measure and compare data from industrial or laboratory assets, that may be subject to mechanical deterioration, or exhibits vibration signatures of interest, in the frequency range from DC to 80 kHz.

The sensor device is built using a wide bandwidth MEMS Accelerometer, ADXL1002 from Analog Devices [24], and a ultrasonic range MEMS microphone, SPH0641LU [25]. This allows for accurate measurements of high frequency vibrations, that may reveal incipient defects in rotating and vibrating components. The use of ultrasound MEMS microphones as a low-cost alternative for condition monitoring by demonstrating their ability to provide accurate and reliable results, at high sample rates, is a feature that is normally only found in high bandwidth piezo accelerometers.

The utilized microcontroller, the Ambiq Apollo3 has a large on-chip SRAM, 384 kB and 1 MB Flash memory, and is utilized due to a desire to perform signal processing and anomaly detection from vibration or acoustic signals on the device. The microcontroller has a consumption of 6 μA/MHz, allowing a significant amount of compute to be performed on the device, rather than spent on transmission of raw data.

The results and project presented may help enable the development of novel low-cost and effective condition monitoring solutions for a variety of applications. The author would like to emphasize the recent emerge of TinyML [26], the ability to perform machine learning (ML) inference on-device, for resource constrained microcontrollers.

The inclusion of both Bluetooth Low Energy (BLE) and LoRa radio in the design in envisioned to support a data collection and ML-training flow through BLE, and a subsequent deployment and inferencing via long range limited bandwidth LoRa, where only result rather than raw data are transmitted.

## 3 Hardware description

### 3.1 Device overview

**Fig. 1:**
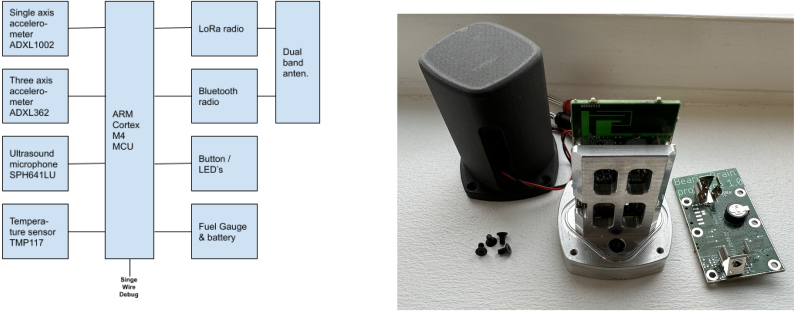
Left: Device block diagram, Right: Assembled sensor without lid mounted and a spare raw assembled pcb, without battery.

The condition monitoring sensor presented herein (see Fig. 1) is intended to allow the user to evaluate and combine sensor readings from an instrumented machine, using the following sensors :

• ADXL1002 single axis Low noise, High frequency accelerometer
• SPH0641LU4H-1 miniature silicon digital microphone with ultrasonic frequency range
• ADXL362 ultralow power, 3-axis MEMS accelerometer
• TMP117 high-precision digital temperature sensor.

The device allows measurement of single axis vibrations up to the app 21 kHz, using the ADXL1002 accelerometer, and up to 80 kHz using the SPH0641 microphone. Additionally the three axis accelerometer, ADXL362, allow simultaneous vibration measurements of up to 200 Hz on three axes. A high precision temperature sensor allows measurement of the ambient temperature .

### 3.2 Microcontroller module

At the heart of the device is a NM180100 combined processor and radio module [27]. The module features an ARM Cortex M4F microcontroller (MCU) from Ambiq semiconductors [28], an Apollo3 Blue, with 1MB flash and 384 kB embedded SRAM. The MCU has builtin Bluetooth Low Energy (BLE) peripheral, and an external Semtech SX1262 LoRa transceiver [29]. The MCU has a builtin 14 bit analog to digital converter to interface the ADXL1002 accelerometer, a PDM peripheral for interfacing the microphone, and a multichannel DMA allowing efficient parallel data collection.

### 3.3 Sensors

The primary sensors are the SPH0641LU microphone [25] and the ADXL1002 accelerometer [24].

The SPH0641LU has a signal to noise ratio of 64.3 dB, acoustic overload (AOP) of 120 dB and a flat frequency response from 100 Hz to 10 KHz. The ultrasonic response from 10 kHz to 80 kHz is not linear, but still useful. The microphone has a pulse density output (PDM), and requires a clock from a PDM master peripheral, available in the MCU.

The accelerometer has a flat frequency response from dc to 11 kHz, and a resonant frequency of 21 kHz. The ADXL1002’s noise density is $25\;\mu g/\sqrt{\mathrm{Hz}}$ when used in ±50 g range, where the sensitivity is 40mV/g. The accelerometer is sampled from the MCU’s 14 bit ADC through a second order aliasing filter with $f_{c}=14.6\;\mathrm{kHz}$ as suggested in [30].

The secondary three axis accelerometer, ADXL362 [31] is connected over SPI, and can measure up to ±8 g It has a resolution of 1 mg/LSB when in ±2 g range and a noise density of $550\;\mu g/\sqrt{\mathrm{Hz}}$.

The TMP117 temperature sensor [32] is a high precision temperature sensor, with a 16 bit readout of temperature with a resolution of 0.0078°C and an accuracy of ±0.1°C. TMP117 is connected over I2C.

### 3.4 Battery and power management

The device can be powered from a single 3.7 v Lithium battery, and is intended to have a Lithium Thionyl Chloride (LiSoCl) battery [33] mounted, if the sensor is used as stand-alone, with wireless transmission of data. LiSoCl batteries, have low pulse current capabilities, but excellent shelf life due to low self discharge, and a high energy density of up app 700Wh/Kg.

A 100mF supercap is present to provide sufficient peak current capability for LoRa radio transmissions, despite the limited pulse current capabilities of the LiSoCl battery. At maximum transmit power, 22dBm, the LoRa transceiver consumes 120mA [29].

A BQ35100 fuelgauge [34] mounted in the I2C bus allows for tracking consumption and the capacity of the battery. Five separate power rails allows shutdown of Bluetooth and LoRa radios, sensors and peripheral flash memory, thus allowing a fine grained power optimization scheme to maximize battery lifetime.

### 3.5 Bluetooth and LoRa

The MCU has a builtin Bluetooth Low Energy (BLE) peripheral, with 94dBm sensitivity and up to 4dBm tx power. BLE allows for short range transmission of raw data, configuration and over the air firmware updates (OTAFWU). The LoRa radio allows for long-range low bandwidth transmission, over LPWAN networks. A Semtech Sx1262 [29] transceiver is fitted in the NM180100 compute module, and has transmission power of up to 22dBm, and provides a sensitivity up to app. 130dBm when deployed in a LoRa network. A full length LoRaWAN transmission may contain 51 bytes in Europe at the lowest datarate DR0. At DR0 a throughput of 250bit/s can be obtained, thus a transmission may endure several seconds. The use of LoRa allows for a low cost infrastructure to be established, with ability to connect to a large number of sensing devices.

### 3.6 RF and antenna

A reference antenna design, optimized for transmitting LoRa at 868MHz and Bluetooth at 2.4GHz is provided by the compute module vendor [27], the antenna and RF routing is implemented to be used on a two layer FR4 fiberglass PCB substrate. An RF connector [35] is populated on the PCB, allowing additional Antenna tuning. Component footprints for additional RF components around the antenna with 0402 passive components is added to the PCB. The footprints are unpopulated or shorted with 0R resistors but allows for additional impedance tuning using a two port network analyzer. Additional tuning may be required depending on the PCB substrate used and may also be beneficial when the device is mounted in an enclosure.

**Fig. 2:**
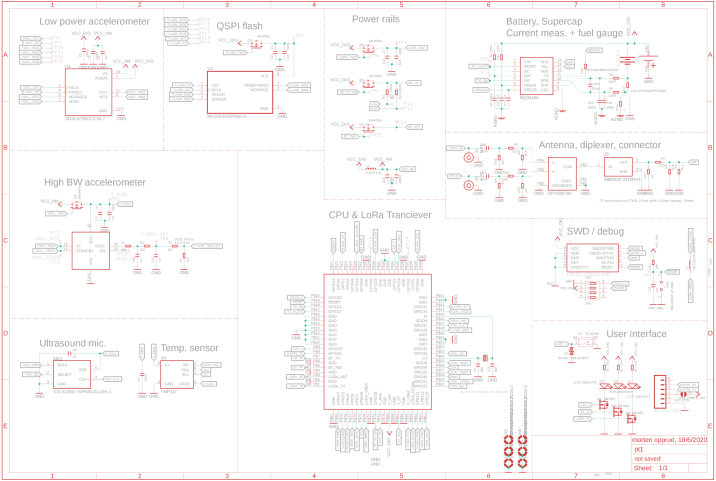
Sensor schematic, the combined microcontroller & radio module is shown as a single component.

The full schematic of the device is shown in Fig. 2.

## 4 Firmware description

The firmware projects presented in this section are intended for evaluation of the hardware, and collection from the individual sensors. The firmware projects are based on Arduino examples, and can be built using the Arduino Integrated Development Environment (IDE) for evaluation of the hardware. The Arduino IDE examples require installation of board support for the Ambiq Apollo3 Microcontroller, install notes are available in the readme.md file in the project repository. For more comprehensive software development the use of Ambiqs Software Development Kit (SDK) is recommended [36]. The use of Bluetooth Low Energy and LoRa for transmission of data is not included in the presented firmware examples, but tutorials are available via sparkfun.com. For LoRaWAN, see the SparkFun expLoRaBLE Hookup Guide, and for Bluetooth Low Energy see the guide development using the Arduino IDE.

### 4.1 SPH641LU serial data readout

The Ultrasonic microphone can be read by configuring the PDM peripheral on the microcontroller. The effective sample-rate of the microphone can be adjusted as outlined in table 522 in the MCU datasheet [28], one has to keep in mind that the amount of available SRAM in the MCU is 784 kB, and sufficient space for a user application may also be required, thus limiting the possible buffer size and sample time that can be achieved. In the Microphone firmware example the sample rate is set to 15625Hz, the adjustable gain of the PDM module is set to 30.0dB and raw data with 16 bit resolution is captured in a 4096 element sample buffer. The PDM driver is interrupt driven and captures samples at the configured sample rate until the sample buffer is full, after this, the data is sent over the serial line with a bitrate of 115.200bps and the sampling is restarted. Table 1 outlines a number of PDM/microphone settings available, by modifying the configuration structure am_hal_pdm_config_t in the program code microphone_serial.ino, the resulting sample-rate and gain setting for the PDM module are shown. The captured data is printed to the serial port, and can be monitored using the Arduino IDEs serial plotting tool, as shown in Fig. 6.

**Table 1:** Microphone sample frequency settings, Fs.

Clock source	Decimation rate	Fs
AM_HAL_PDM_CLK_750KHZ	24	15.625 Hz
AM_HAL_PDM_CLK_6MHZ	64	46.875 Hz
AM_HAL_PDM_CLK_12MHZ	60	100.000 Hz

### 4.2 ADXL1002 serial data readout

The ADXL1002 accelerometer is connected to the MCUs 14 bit analog to digital converter. In the ADXL1002 test application adxl1002_serial.ino the program loop reads the ADC and transmits the data over the serial port at 115.200bps, resulting in an effective samplerate of app 16kHz. This program does not use interrupts to control the sample rate. However the MCUs timer/counter3 can be configured to trigger the ADC at a fixed sample rate, where an interrupt may be used to store data in a buffer before transmission, similar to the microphone firmware example.

### 4.3 ADXL362 serial data readout

The ADXL362 is connected over SPI. In the adxl362_serial.ino example the ADXL362 is configured in ±2 g range with an internal sample rate of 100Hz. X, Y and Z data are read via SPI when new samples are present, and sent over the serial port with a bit rate of 115.200bps. The test application is using the accelerometer in polled mode. The ADXL362 can be configured in great detail to provide interrupts, for instance when a threshold in X, Y or Z is detected. An embedded 512 sample FIFO in the ADXL362 allows additional motion event detection, or power savings if the MCU is put to sleep, and woken by an interrupt when the FIFO is filled above a given threshold, and thus only need to initiate data reads from the device periodically. The ADXL362 application requires installation of an external driver library, see [37] (see Table 2).

**Table 2:** Design files of the sensor.

Design filename	File type	Open source license	Location of the file
**Hardware**			
pt1.sch	Schematic	CC BY-SA 4.0	osf.io/kn9rx
pt1.brd	PCB	CC BY-SA 4.0	osf.io/6duks
pt1.lbr	Library	CC BY-SA 4.0	osf.io/jtxds
pt1_BOM.txt	Bill of material	CC BY-SA 4.0	osf.io/7c8ez
**Enclosure**			
pt1_foot.stl	CAD drawing	CC BY-SA 4.0	osf.io/jhva6
pt1_mount.stl	CAD drawing	CC BY-SA 4.0	osf.io/g4avm
pt1_lid.stl	CAD drawing	CC BY-SA 4.0	osf.io/qyu58
**Firmware**			
microphone_serial.ino	source code	CC BY-SA 4.0	osf.io/x2h5v
adxl1002_serial.ino	source code	CC BY-SA 4.0	osf.io/gy4ep
adxl362_serial.ino	source code	CC BY-SA 4.0	osf.io/tfvkn

## 5 Design files

### 5.1 Design files summary

For the *enclosure*, the foot and pcb-mount are manufactured in aluminum, on a CNC machine, the lid is 3D printed in plastic, PLA or similar. The entire design can be 3D printed, but Aluminum is preferred, for the foot and pcb-mount due to temperature stability, and a higher stiffness for transmitting vibrations to the sensors.

The hardware has been designed using Autodesk eagle (free version), version 9.62.

Firmware for the sensor is implemented using Arduino IDE, v 2.0.3

Segger Jlink-EDU is used for programming the microcontroller.

Solidworks was used for designing the mechanical enclosure.

The design files are summarised in table Table 2.

## 6 Bill of materials

**Table 3:** Bill of materials (BOM).

Designator	Component	Number	Unit cost ($)	Total cost ($)	Source of materials
**Hardware**					
	Circuit board	1	6	6	Eurocircuits.com
	Electronics	1	150	150	digikey.com
	NM180100	1	25	25	digikey.com
	Battery LS14500	1	10	10	tme.eu
**Enclosure**					
	M3 × 10 screw, countersunk	6	0.5	3	digikey.com
	M3 × 6 screw	4	0.5	2	digikey.com
**Misc**					
	Stencil for solder paste	1	15	12	Eurocircuits.com
	USB-Uart cable	1	20	20	ftdichip.com
	Jlink-edu programmer	1	35	35	segger.com

### 6.1 Bill of materials summary

The components used to populate the PCB are not listed in the table above. The bill of material list for electronic components *pt1_BOM.txt* is available on the project file repository. A bill of material that can be uploaded directly to a *digikey.com* cart is found in Table 3, the compute module need to be ordered separately, a link is also provided in Table 3.

## 7 Build instructions

### 7.1 Tools

The following tools are recommended for the build:

• Solderpaste, lead free.
• 18 mm snap-blade for cutting knife or spatula for solder paste dispersion.
• Re flow solder oven.
• Fine-tip tweezers.
• Vacuum-pen.
• Clamps, 2 pce. (for stencil fix, if not using stencil/pcb templates)

### 7.2 Procurement of parts

The parts listed in the Bill of materials *(pt1_BOM.txt)* parts can be purchased from electronic retailers such as Mouser, Farnell, or Digikey. A link to a shopping cart with direct links is provided in the project repository. Passive components can be substituted with alternative parts. Using at least 1% thin-film resistors is recommended, and 10% 5V X7R or C0G ceramic capacitors when possible. Active components cannot be substituted without redesigning the circuit.

### 7.3 PCB fabrication

The circuit board is a two layer PCB, made from 1.55 mm FR4 core, with top and bottom copper layers of 0.018 mm. The boards and stencil for dispensing solder paste has been manufactured and purchased at eurocircuits.com, additionally eurocircuits EC-stencil-fix are used to align and center PCB’s during application of solder-paste. The PCB’s are manufactured in panels of four. Alternative PCB manufacturers such as jlcpcb.com or pcbway.com maybe also be considered. Pay attention to RF characteristics of antenna and RF track routing if different material, copper or substrate thickness is used. A two layer PCB is used to keep cost and design complexity at a minimum. The cost of a single PCB is app 6$ for an order of 5 panels with 4 PCBs at eurocircuits.

### 7.4 Board assembly

**Fig. 3:**
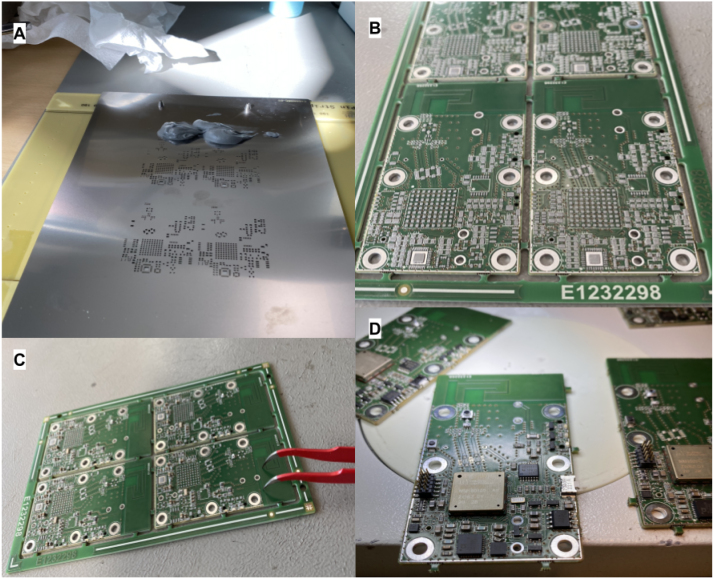
PCB assembly, **A**:Solder paste application, **B**: Raw PCBs with solder paste applied, **C**: Passive components placed, **D**: Boards soldered.

The boards can be assembled manually as shown in Fig. 3, since only a few RF tuning components are in 0402 format, the remaining passive components are mostly 0603 and 0805. Application of solder paste should be done with a stencil purchased with the PCBs, hereafter components can be picked and placed with fine-tip tweezers and vacuum pen for larger IC’s. The boards should be soldered in a reflow oven, with adjustable temperature profile, to minimize thermal stresses of components. Alternatively, a hot air soldering station could be used though this method does not allow the temperature to follow the recommended profile. Thorough solder inspection under an microscope or magnifying glass should be performed before applying power to the circuit board.

#### 7.4.1 Hardware modification, first board revision

In the hardware design, available on the projects repository, a small workaround needs to be implemented on the board, if using the design “as is”, the user also has the option to modify the design, and fix the issue in the PCB/schematic layout. The problem is caused by two signals SWDIO and ADXL_STDBY being swapped. The ADXL1002 will still function with ADXL_STDBY disconnected, but the MCU cannot be programmed without SWDIO properly connected.

The minimum workaround requires cutting the connection from J3 pin2, near the debug connector, and cutting the connection to the accelerometer, ADXL_STDBY signal, near the ADXL1002. A thin wire can the be connected from J3 pin2, the SWDIO signal, to testpad TP23, which is connected to the microcontrollers pin GPIO21, where the SWDIO signal to the microcontroller is available.

The workaround is best performed by cutting the required tracks before mounting and soldering components, and soldering the thin wire in place after the surface mount (SMD) components are fitted and soldered.

**Fig. 4:**
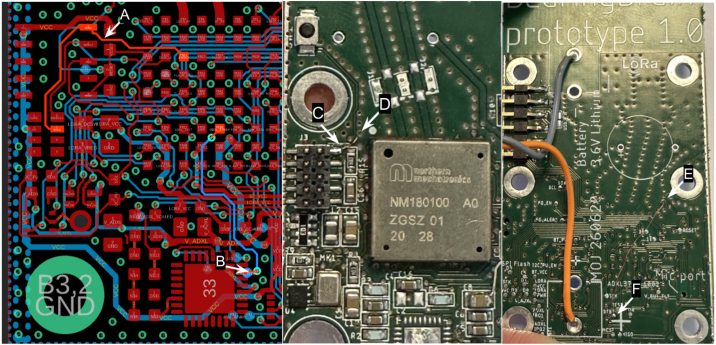
Hardware Modification Left: **A** and **B** shows the PCB tracks that are to be swapped, Mid: **C** and **D** shows solder and mount of wire, Right: **E** and **F** shows wire on backside of PCB, soldered onto testpoint ADXL_STDBY.

To perform the workaround, follow the steps below, as illustrated in Fig. 4

• A - Cut the signal wire SWDIO just right of the J3 connector on the PCB’s top (component) layer
• B - Cut the ADXL_STDBY signal on the top PCB layer, just outside the ADXL1002 IC footprint
• C - Cut a very thin wire, app 10 cm. Shown in the figure is an insulated copperthread. Solder this onto J3 pin2
• D - Gently pull the wire through one of the plating holes, shown here is an GND plating, just next to the J3 connector
• E - Cut the wire in an appropriate length so it can reach the testpin (TP23), labeled ADXL_STDBY marked with F
• F - Solder the wire onto the ADXL_STDBY (TP23) testpoint

### 7.5 Board bring-up

Before connecting power to the board, ensure that all components are properly soldered and no solder bridges exists between pins on ICs.

**Fig. 5:**
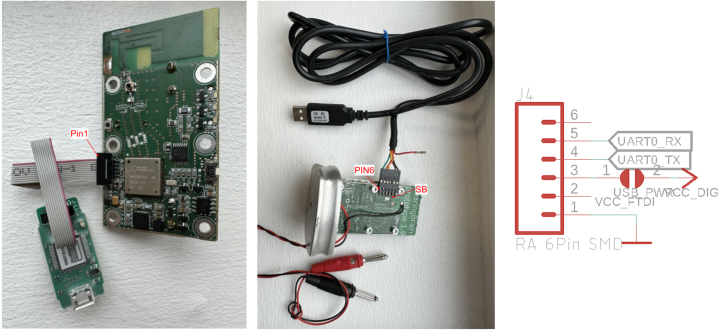
Left: Jlink programmer connected, pin1 on PCB shown, Middle: USB-serial cable connected for data capture and wires for external power supply, Right: J4, connector for serial data transfer - pin mapping, and solder jumper for power via USB serial cable.

1. Connect test wires to the battery terminals, marked **‘+’** and **‘-’** on the non component side of the board
2. Use a laboratory power supply with current limiter, set the current limit to app 50mA and the voltage to 3.3v, and connect the test wires.
3. The board should use less than 10mA when powered up without firmware, turn off the power supply.
4. Attach the SWD programmer, ensure that pin1 on the programmer connects to pin1 on the sensor board (see Fig. 5)
5. Reconnect the board to power, connect the SWD programmer, and program one of the compiled applications, described under Firmware description into the MCU.
6. Press reset, current consumption should be app 5–10mA, and the red LED D3 should light up.

## 8 Operation instructions

In the following, it is assumed that one of the firmware projects microphone_serial.ino, adxl362_serial.ino or adxl1002_serial.ino has been programmed into the MCU. Brebuilt binary files are available in the firmware/prebuilt folder in the project repository.

**Fig. 6:**
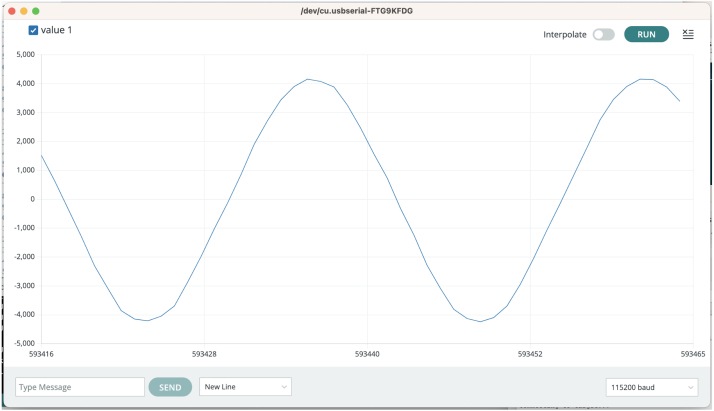
Operation of the microphone validation firmware with a 400 Hz sine wave sound played near the microphone.

1. Powering the board, this can be done in one of three ways
  • Through a 3.6 V LiSoCl AA sized battery on the battery clips.
  • Through a 3.3 V powersuppply to the battery terminals.
  • Via a USB serial cable with 3.3 V supply. Please note, some cables e.g. FTDI TTL-232R-3V3 are 3.3 V compatible in RX/TX lines, but provide **5V VCC and must NOT be used**, where other models supply 3.3 V, e.g from sparkfun. If this option is used, solder the solder-bridge marked **SB** in Fig. 5.
2. Connect a USB to serial, 3.3 V compatible cable to TX/RX/GND pins, as shown in Fig. 5.
3. Open a terminal program at 115.200bpb, use default 8N1 configuration, alternatively open the *Arduino Serial Monitor* to see the displayed wave forms.
4. Turn on power to the board.
5. Data should now be visible/printed in the selected terminal program, Fig. 6 shows a 400 Hz sine wave signal, generated from a speaker, near the microphone.

## 9 Validation and characterization

### 9.1 System characterization

**Fig. 7:**
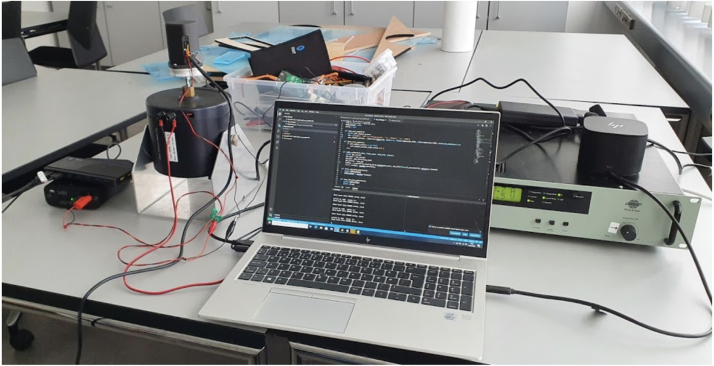
Validation setup, from left to right showing: power supply, shaker table with sensor device mounted, PC for executing test-script and generation of audio signal to amplifier, power amplifier.

To validate the sensors ability to measure vibration in the operating spectrum, a fully assembled sensor is installed on a shaker table and exited. The sensor is mounted on a shaker table that is capable of generating vibrations in one direction. The mount is mechanical, using a threaded bolt, screwed into the shaker tables brass mount, see Fig. 7.

The shaker table is a type 4809 [38], driven by a power amplifier type 2718 [39] from Bruel & Kjaer. The frequency range of the shaker table is from 10 Hz to 20 khz, and with a payload of app 250 g from the assembled sensor with battery and enclosure, the maximum excitation possible is around 15 g.

The accelerometers sensing are directional, where the microphone is not. The excitation of the microphone will depend on the ability of the enclosure to conduct sound, and may be affected by interference occurring inside the enclosure, as vibrations excite the circuit board and enclosure. As a result no direct relation between acceleration in g and sound pressure level (SPL) captured by the microphone can be established, however a good correlation among acceleration and SPL is observed.

Four test sequences are used to characterize the sensors ability to measure vibrations. Two low frequency sweeps allows comparison of the ADXL362 and ADXL1002 accelerometers from DC up to 200 Hz. Additional two high frequency sweeps from 1-20 kHz allows comparison of the ADXL 1002 accelerometer with the SPH0641LU microphone, as the microphone is not expected to perform well under 100 Hz. A low intensity sweep is used to evaluate the signal to noise ratio of the measurements, where the high intensity allows for comparison of the measured data sample rate, by comparing the frequency content after performing a Fast Fourier Transform on the raw data. Each test sequence capture one second of raw data at the samplerate outlined in Table 5.

Four validation tests are conducted, as outlined in Table 4.

The three axis accelerometer ADXL362 can be used in ±2 g, ±4 g, ±8 g ranges, this is controlled by firmware. The ±4 g range is used for the validation tests, with an output data rate of 400Hz, thus a signal of 200Hz can be measured.

**Table 4:** Validation parameters.

Validation sequence	Frequency range	Vibration amplitude
1: Low amplitude, Low Frequency	10–200 Hz in steps of 25 Hz	Low
2: High amplitude, Low Frequency	10–200 Hz in steps of 25 Hz	High
3: Low amplitude, High Frequency	1200–19200 Hz in steps of 1000 Hz	Low
4: High amplitude, High Frequency	1200–19200 Hz in steps of 1000 Hz	High

ADXL1002 has a fixed ±50 g measurement range with 40mv/g sensitivity, and is sampled by the MCU’s analog to digital converter with a sample rate of 40kHz. The pulse density signal from SPH0641LU is demodulated by the MCUs PDM peripheral which has a programmable gain from −6dB to 40.5dB in steps of 1.5dB. In the validation tests, a gain of 30dB and a sample rate of 100kHz is used. The sensor settings are summarized in Table 5.

**Fig. 8:**
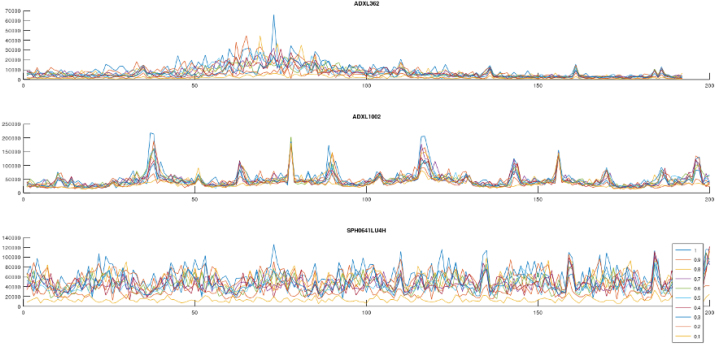
Sensor validation, ADXL362, ADXL1002 and SPH641U on shaker table Low amplitude 0–200 Hz.

**Fig. 9:**
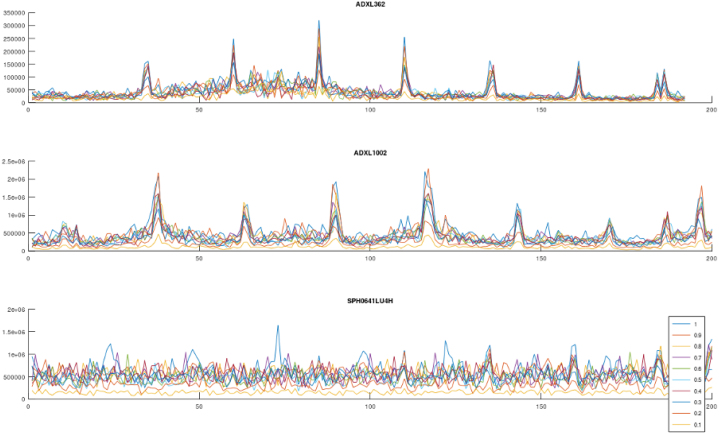
Sensor validation, ADXL362, ADXL1002 and SPH641U on shaker table High amplitude 0–200 Hz.

**Fig. 10:**
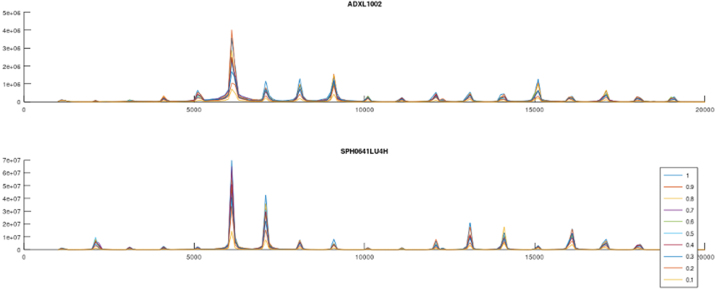
Sensor validation, ADXL1002 and SPH641U on shaker table Low amplitude 0–20 kHz.

**Fig. 11:**
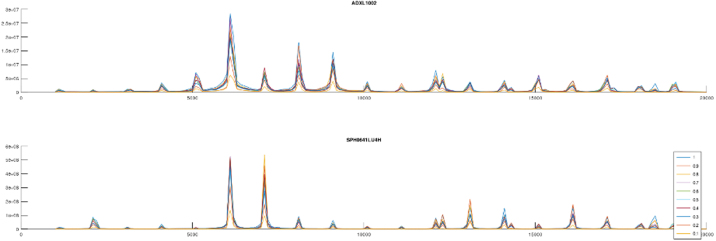
Sensor validation, ADXL1002 and SPH641U on shaker table High amplitude 0–20 kHz.

For each test sequence a set of fixed frequencies are applied to the shaker, as listed in Table 4, data is captured for 1 s for each frequency, from each sensor. Each frequency is applied at amplitudes ranging from 0.1 to 1.0 increasing 0.1 for each iteration. A Fast Fourier Transform (FFT) is performed on each data set, and the corresponding signals are plotted in a single plot. This is repeated for each validation sequence and visualized in Figs. 8, 9,10, 11 .

**Table 5:** Sensor sensitivity settings for validation.

Sensor	Sample rate	Note
ADXL362	400 Hz	±4 g range
ADXL1002	40 kHz	±50 g range (fixed)
SPH0641LU	100 kHz	30 dB gain mode

The ADXL1002 performs well in both high and low frequency range, and shows a better signal to noise ratio than the ADXL362 as indicated in Fig. 8. The ADXL362 has an internal oscillator for sampling the internal 3 axis MEMS sensor, and some deviation in the obtained frequency in comparison with ADXL1002 can be seen in Fig. 9. This corresponds to the datasheet for the ADXL362 [31] that outlines a possible clock frequency deviation from ideal output datarate of up to 8%.

The response from the SPH0641LU microphone is plotted against the ADXL1002 and reveals some mechanical eigenfrequencies in the PCB and mechanical design in particular from 6 kHz to 9 kHz. In addition, a correlation plot, Fig. 15 displays the calculated correlation coefficient r among the directional acceleration and audible vibration signals captured by the microphone. The microphone may capture up to 80 kHz, but the shaker table used for validation is limited to 20 kHz.

### 9.2 Detection of a bearing defect

**Fig. 12:**
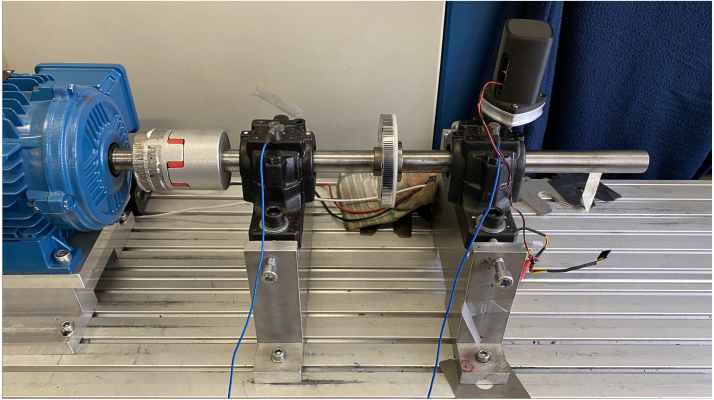
Test rig with two SKF2206 bearings driven by a speed controlled motor, the bearing on the right has an outer ring damage, the prototype is installed using a threaded adapter in a lubrication port of the bearing pillow block.

In addition to the characterization of the frequency response from the Microphone and Accelerometers, in the previous chapter, a validation of the sensor is performed by installing the sensor on a bearing test-rig with a manufactured outer ring defect present in one bearing. The bearing under test is a dual row self aligning ball bearing, type SKF2206EKTN9 [40], with an outer ring defect manufactured using electro discharge machining (EDM) with a size of 5 × 0.5 × 0.25 mm. The installed sensor and test rig is illustrated in Fig. 12.

As the balls in a bearing excite surrounding mechanical structures, when they roll over an infant bearing defect, a high frequency “ringing” is often seen. The observed frequency of this periodic signal is determined by the eigenfrequencies of the bearing components. In addition the increased sensitivity of accelerometers around their resonance frequency, though this is beyond the linear response of the sensor, is often used to pick up early bearing defects.

In a ball bearing, a number of characteristic frequencies $F_{o}$, $F_{i}$, $F_{r}$ and $F_{c}$ are typically monitored to detect bearing defects, these are determined by the bearings geometric parameters, and the rotating speed of the application. The reader is referred to [2] for an introduction to bearing defect analysis.

**Fig. 13:**
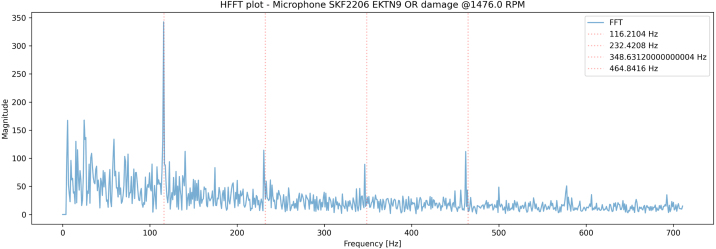
Enveloped FFT plot of the microphone signal from the bearing test-rig. The spectrum from 0-700 Hz is shown and indicates the presence of an outer ring defect, as $F_{o}$ and its harmonics are visible.

**Fig. 14:**
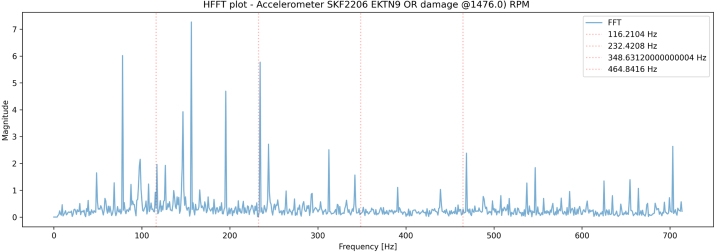
Enveloped FFT plot of the accelerometer signal from the bearing test-rig. The spectrum from 0-700 Hz is shown, also indicating the presence of a lot of spectral noise, but the presence of an outer ring defect, as $F_{o}$ and its harmonics are visible.

In Fig. 13, Fig. 14 one second samples from the Microphone with Fs=100kHz and the accelerometer with Fs=40kHz are captured from the bearing, with a manufactured outer ring defect (Fig. 12), subsequently the envelope of the signal is found using Hilbert transform, before performing FFT and plotting the results.

Both plots indicate the presence of the outerring defect, where the accelerometer captures a significant amount of spectral signals, in addition to the defect.

**Fig. 15:**
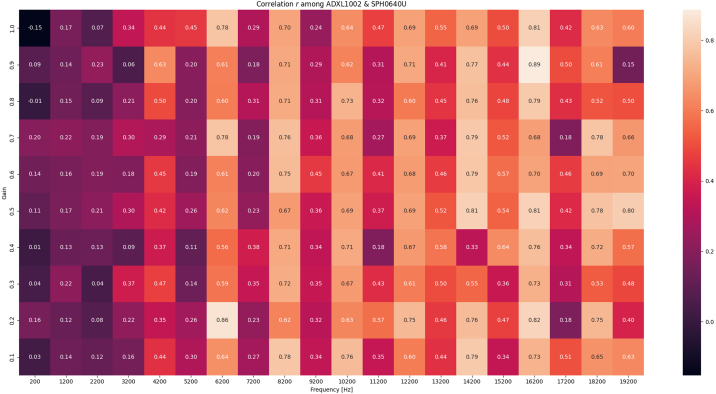
Signal correlation for each 1 KHz spaced excitation frequency captured from the SPH0641U microphone and the ADXL1002 accelerometer. X axis show excitation frequency and Y axis, excitation amplitude (Validation sequence 4).

## 10 Conclusion

The advancements of capabilities within the field of MEMS based sensors has paved the way for the development of low power and low cost sensing devices.

In this work, the design of a low cost condition monitoring sensor, with a wide measurement bandwidth, has been presented. The device has been validated on a shaker table, to compare vibration signals from accelerometers and microphone. In addition the sensor has been installed on a test-rig with a defect bearing, where the presence of an outer-ring defect has been established from captured audio and vibration signals.

The available compute capabilities and reasonable amounts of RAM and Flash memory offered by low cost 32 bit micro-controllers, like the Ambiq Apollo 3 utilized in this project, allows the user to implement significant on-device signal processing and possible advanced machine learning or anomaly detection algorithms on the device. Through one of the two wireless radio standards implemented LoRa and BLE, results from calculations or raw data may be transmitted wireless off the device, so it may participate in industry 4.0 networks.

## Ethics statement

The work does not use any human or animal subjects.

## CRediT authorship contribution statement

**Morten Opprud Jakobsen:** Conceptualization, Data curation, Formal analysis, Funding acquisition, Investigation, Methodology, Project administration, Resources, Software, Validation, Visualization, Writing – original draft, Writing – review & editing.

## Declaration of competing interest

The authors declare that they have no known competing financial interests or personal relationships that could have appeared to influence the work reported in this paper.
